# Bradykinin increases resensitization of purinergic receptor signaling in glioma cells

**DOI:** 10.1186/1475-2867-10-35

**Published:** 2010-09-27

**Authors:** Héctor E López-Valdés, Luis Beltran-Parrazal, Kevin C Brennan, Andrew C Charles

**Affiliations:** 1 Department of Neurology, David Geffen School of Medicine, University of California, Los Angeles, CA, USA; 2 Program of Neurobiology, Universidad Veracruzana, Xalapa, Veracruz, México

## Abstract

**Background:**

Purinergic receptor-mediated signaling plays an important role in the function of glial cells, including glial tumor cells. Bradykinin is also an important paracrine mediator which is highly expressed in brain tumors and may correlate with their pathological grade. Interaction between bradykinin and purinergic signaling may therefore be involved in the regulation of glial tumor cells.

**Results:**

We examined the effect of bradykinin on glial purinergic signaling in an immortalized glioma cell line. Confocal calcium imaging revealed that ATP evokes an increase in [Ca^2+^]_i _in the U87 human astrocytoma cell line. This response was reduced with repetitive application of ATP, likely due to receptor desensitization. However exposure to bradykinin increased the Ca^2+ ^response to a second application of ATP, consistent with increased resensitization. The bradykinin effect on resensitization was similar in the absence of extracellular Ca^2+ ^or in the presence of the PKC activator PMA, but was inhibited by the protein phosphatase inhibitor okadaic acid and the PI3K inhibitor LY294002.

**Conclusions:**

Modulation of protein phosphatases and the PI3K pathway may represent a mechanism by which bradykinin potentiates purinergic signaling in glial cells.

## Background

ATP is a primary extracellular signaling molecule for glial cells in the CNS [1,2]. In astrocytes, ATP is a key messenger for the intercellular communication of calcium waves, in which increases in [Ca^2+^]_i _propagate from cell to cell across multiple cells [3-5]. Glial cell calcium waves have been characterized extensively *in vitro *in a variety of different tissue preparations, and also more recently *in vivo *in rodent cortex and retina [6-10]. They are thought to play physiological roles in the modulation of neuronal activity and vascular function, in addition to contributing to pathological processes such as cortical spreading depression and seizures [11,12]. Purinergic signaling is also believed to play an important role in the development and proliferation of glial cells under both physiological and pathological conditions, including those associated with glial tumors [13-15].

Glial cells respond to ATP through P2 purinergic receptors that belong to two families: P2Y G protein-coupled receptors (GPCR) and P2X ligand gated ion channels. Activation of P2Y purinergic receptors triggers G-protein mediated activation of phospholipase C γ (PLC_γ_) and increases levels of inositol 1,4,5-triphosphate (IP_3_) and diacylglycerol (DAG), leading to elevations in intracellular calcium concentration and the activation of protein kinase C (PKC). By contrast, activation of P2X purinergic receptors leads to an increase in intracellular calcium concentration by influx of extracellular calcium through the receptor channel. In glial cells, the sustained increase in [Ca^2+^]_i _evoked by ATP is mediated predominantly via activation of P2Y purinergic receptors, although the response to higher concentrations of ATP may also involve Ca^2+ ^influx through P2X receptors [1].

Activation of GPCRs by agonists not only results in the G protein- dependent activation of the effector system, but also triggers coordinated molecular mechanisms governing the ongoing response of the receptors to further stimulation [16,17]. GPCR receptors show attenuation or loss of responses by repetitive agonist exposure, referred to as desensitization. Reduction of GPCR responsiveness to an agonist over time represents an important physiological feedback mechanism that protects against both acute and chronic receptor overstimulation. After a period of desensitization, receptors recover their responses to agonists (resensitization), which enables receptors to maintain their ability to respond to agonists over time [17].

GPCR desensitization involves multiple distinct events including the uncoupling of receptors from their G proteins, the internalization and sequestration of receptors to endosomes, and down-regulation [16]. Receptor G protein uncoupling in response to receptor phosphorylation is the most rapid means of attenuating GPCR responsiveness and occurs within seconds to minutes following agonist activation. Phosphorylation is mediated by two families of protein kinases: the second messenger dependent protein kinases (e.g. PKA, PKC) and the G protein-coupled receptor kinases GRPKs; [18]. Receptor sequestration is also initiated within seconds to minutes of receptor activation and potentially contributes to receptor desensitization by limiting the number of plasma membrane accessible receptor binding sites. Down-regulation, a decrease in the total cellular complement of GPCRs, occurs in response to longer-term exposure to agonist from minutes to hours [18]. Resensitization of receptors involves the reversal of these processes, namely receptor dephosphorylation by phospatases, recovery of sequestered receptors to the plasma membrane, and increased synthesis and or trafficking of receptors to their sites of function [17].

Bradykinin is a nonapeptide (or kinin) formed from precursors (kininos) through actions of plasma and tissue kallikreins [19]. Kinins are implicated in physiological and pathological processes such as vasodilatation and inflammation [19]. Two kinin-specific GPCR have been reported, B1R and B2R. The B1R mediates the actions of Lys-des-Arg^9^- bradykinin whereas B2R is activated by the main kinin, bradykinin [19,20]. Activation of B2R is preferentially coupled to G proteins of the Gα-q subtypes, which in turn activate PLC_β_, leading to production of IP3 and release of intracellular calcium [19,20]. B2R activation also activates PKC, phosphatase A2, phospholipase C, and phosphoinositide 3-kinase (PI3K), and stimulates production of nitric oxide [19,20]. B2R's are expressed in many different cells including astrocytes [19-25] and human astrocytic tumors [26,27]. It has been reported that B2 receptors are more highly expressed in glioma cells as compared with normal astrocytes [28] and the level of expression of B2 receptors may be correlated with the grade of human gliomas [29].

Bradykinin and ATP-mediated signaling may interact via multiple pathways. Bradykinin potentiates the ATP response in Xenopus oocytes that express P2X receptors [30]. Meanwhile, ATP causes a desensitization of B2R in a neuroblastoma cell line [31] and bradykinin promotes/induces ATP release from astrocytes [32]. In this work, we explore the effects of bradykinin on purinergic receptor signaling in the U87 human astrocytoma cell line. We show that bradykinin promotes resensitization of P2Y purinergic receptors via activation of PI3K and the phosphatase A2 pathways.

## Methods

### Cell culture

Human astrocytoma U87 cells were grown in DMEM/F12 mixture (50-50) medium supplemented with 10% (V/V) fetal calf serum, 100 units/ml penicillin, and 10 mg/ml streptomycin in humidified air with 5% CO_2 _at 37°C. 1 × 10^6 ^cells were grown to sub-confluent density on Poly-D-lysine treated round glass cover slips (18 mm diameter) for 4-6 days prior to experimentation.

### Measurement of [Ca^2+^]_i_

Cells were loaded with 5 μM fluo-4-AM for 20 min at room temperature in Hanks Buffered Saline Solution (HBSS) with HEPES (pH 7.38) that was used as the standard experimental solution. All experiments were performed with constant perfusion (2 ml/min) at room temperature (22°C) on a Nikon Diaphot microscope coupled to a custom confocal imaging system as previously described [33]. Briefly, excitation from a 475 nm diode laser was delivered via scanning mirrors to the specimen through a 40× lens. Fluorescence emission was gathered through a dichroic mirror and 535 bandpass filter to a photomultiplier tube (Hamamatsu) and images were acquired at 1 Hz for 6 minutes by an image acquisition board (Bitflow Raven) controlled by Video Savant software. Before application of test substances, cells were continuously perfused for 10 minutes with standard solution. Ligands were added for the duration specified by continuous perfusion. For dose-response relationships of bradykinin, the compound was applied for 5 minutes.

### Bradykinin Pretreatment and Repetitive ATP application

After exposure to either control solution or 200 nM bradykinin for 5 minutes, cells were then washed for 10 minutes with HBSS-HEPES, after which ATP (10 μM) was applied for 1 minute, ATP was washed off for 2 minutes, followed by a second 1-minute application of ATP. The PKC activator PMA (1 μM), the BK2R antagonist HOE-140 (1 μM), and the phosphatase and kinase inhibitors (Okadaic acid, 40 nM and LY294002, 50 μM, respectively) were applied for 10 minutes prior to application of bradykinin. All chemicals were dissolved in HBSS-HEPES. For experiments with Ca^2+ ^free medium, ATP was applied in HBSS-HEPES containing no added Ca2+, after which cells were returned to normal HBSS-HEPES.

### Analysis

Regions of interest (30 × 30 pixels) were placed in the center of the cell body of every single cell in each microscopic field, and fluo4 fluorescence vs. time was determined for each cell using ImageJ and Origin 6.0 software (Northampton, MA, USA). Ca^2+ ^responses were characterized based on ΔF/F_o _calculated as (F_1 _- F_0_)/F_0_, where F_1 _is the fluorescence at a given time and F_0 _is the basal mean fluorescence 1 minute before of application of test substances. Peak Δ/F_o _was determined for each individual cell for each ATP application, and these peak responses were compared for individual cells to determine the extent of resensitization. The area under the curve (AUC) for each [Ca^2+^]_i _response was also quantified using Origin software and values for AUC were also compared for the 1^st ^and 2^nd ^ATP response as an indication of resensitization. The ratio of the peak Δ/F_o _or AUC for the 2^nd ^vs. 1^st ^response to ATP was designated as the % resensitization for each cell, and these values were averaged for all cells. A sigmoidal dose-response curve was fitted by Origin 6.0 (Northampton, MA, USA). Statistical analysis was performed using GraphPad Prizm 4.0 (La Jolla, CA, USA). Data are presented as mean ± SEM; p < 0.05 was considered significant.

## Results

### Bradykinin [Ca^2+^]_i _response

**Figure 1:**
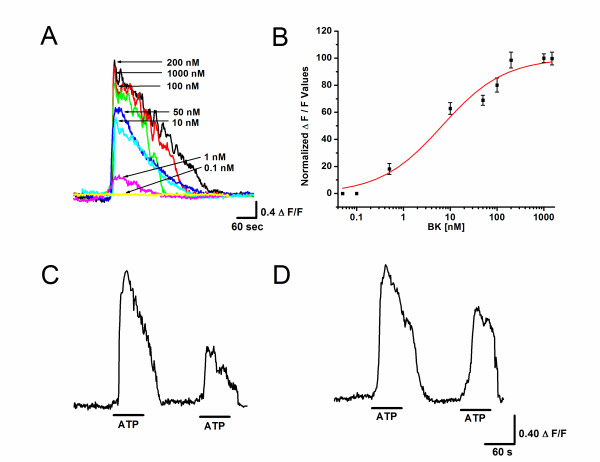
**Response of U-87 cells to Bradykinin**. (A) Superimposed line traces show the calcium increase upon exposure to different concentrations of bradykinin. The traces are the fluo4 ΔF/F vs. time for representative cells. (B) Dose-response relationships of bradykinin. Each value represents the average peak value of ΔF/F from 78-276 cells for each concentration tested. The average peak values for each concentration were fitted with sigmoidal dose-response and EC50 obtained was 6.7 nM of bradykinin. The maximum response was reached with 200 nM. (C) Typical Ca^2+ ^response to repetitive application of ATP (10 μM). Line trace shows fluo4 ΔF/F vs. time for a representative cell. Note that the amplitude of the increase in [Ca^2+^]_i _in response to the second application ATP is significantly reduced as compared with the first application. (D). Typical Ca^2+ ^response to ATP in a cell pre-treated with bradykinin (200 nM). Pre treatment with bradykinin did not results in any significant change in the response to fist exposure to ATP, however, the second response to ATP showed an increase in the amplitude as compared with untreated cells (C).

Since activation of B2R leads to release of intracellular calcium [19,20], we first determined the concentration that evoked the maximum [Ca^2+^]_i _response in U-87 cells. Results from those experiments (Figures 1A-B) showed that maximum averaged peak response reached a plateau at 200 nM of bradykinin, we therefore used this concentration to investigate the effects of bradykinin on the response to ATP. [Ca^2+^]_i _was monitored for 10 minutes following bradykinin exposure. After the initial response to bradykinin, no further changes in [Ca^2+^]_i _were observed over the next 10 minutes.

### Desensitization and Resensitization of ATP receptors

GPCR, including P2Y purinergic receptors, undergo desensitization and resensitization after agonist exposure [18]. We investigated whether bradykinin can affect these processes. When U-87 glioma cells were exposed to repetitive 1 minute application of ATP (10 μM) separated by two minutes, the response for the second application of ATP showed a reduced [Ca^2+^]_i _response (Figure 1C). Cells incubated with bradykinin (200 nM for 5 minutes) and then exposed to the same double application of ATP, ten minutes later, also showed a reduced [Ca^2+^]_i _response. However, the peak and the AUC for second response to ATP were significantly (p < 0.001) larger than in control cells (Figure 1D), suggesting that previous exposure to bradykinin significantly increased the resensitization of P2Y receptors.

**Figure 2:**
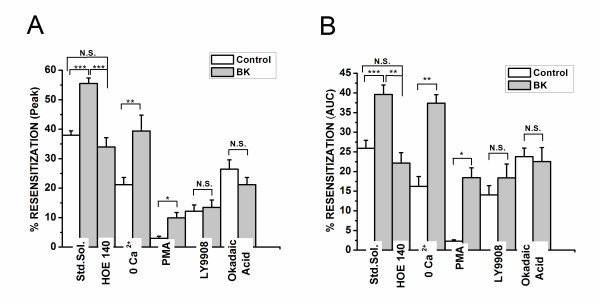
**Mechanisms of bradykinin effects on ATP resensitization**. Graph A shows the average (± SEM) of ratio of the peak Ca^2+ ^response and graph B shows the average (± SEM) ratio of the area under the curve (AUC) of the Ca^2+ ^response of the second ATP response for all conditions tested. The ratio of the peak Δ/F_o _(A) or AUC (B) for the 2^nd ^vs. 1^st ^response to ATP was designated as the % resensitization for each cell, and these values were averaged for all cells. Statistical significance was obtained using one way ANOVA with post-hoc Turkey. ***p < 0.001, **p < 0.01 and *p < 0.05, denotes statistic significance. Note that the enhancement of resensitization persisted on removal of extracellular Ca^2+^, but is lost on inhibition of phosphatases with okadaic acid, and by inhibition of PI3 kinase with LY294002.

To confirm that the activation of BK2R is necessary for the increase in the resensitization of the ATP receptors, we applied the selective BK2R antagonist, HOE-140 (1 μM) concurrently with bradykinin application. Cells treated with HOE-140 plus bradykinin showed no differences in the responses to ATP with respect to the control cells (standard solution); moreover, the evoked increase in the resensitization of the second response to ATP observed in the group with bradykinin alone was significantly blocked (p < 0.001, Figures 2A-B)

Application of ATP (10 μM) consistently evoked an increase in [Ca^2+^]_i _in U87 cells. This response was blocked by pretreatment with the PLC inhibitor U73122 (10 μM) and was not inhibited by the removal of extracellular Ca^2+^, indicating that the response was mediated primarily by G-protein coupled purinergic (P2Y) receptors as has been previously described [1].

**Figure 3:**
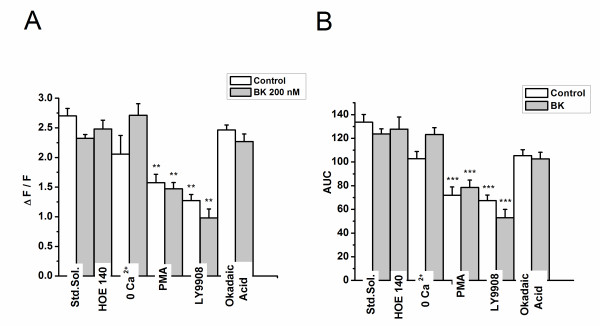
**The first ATP response in bradykinin-treated vs**. **untreated cells**. A and B graphs shows the average ratio (± SEM) of the peak Ca^2+ ^response and the average (± SEM) of area under the curve (AUC) of the Ca^2+ ^response of the first ATP response for all conditions tested (n = 47-276 cells per condition). Statistical significance was obtained using one way ANOVA with post-hoc Turkey. ***p < 0.001, **p < 0.01 and *p < 0.05, denotes statistic significance compared with control.

To examine the contribution of extracellular calcium to the ATP resensitization, similar experiments were performed in Ca^2+ ^free medium. When ATP was applied in 0 Ca^2+ ^medium, the amplitude of the first ATP response was not significantly different from those observed in normal Ca^2+ ^medium (figures 3A-B), suggesting that the response was due to release of Ca^2 ^from intracellular stores. The increase in resensitization we observed with bradykinin application was also preserved in 0 Ca^2+ ^medium, although a significantly smaller peak amplitude (39% average resensitization in 0 Ca^2+ ^vs. 55% average resensitization in normal Ca^2+ ^medium, Figure 2A, p < 0.001). These results indicate that Ca^2+ ^influx is important for the resensitization of the response to ATP, likely in part by replenishing intracellular Ca^2+ ^stores. However, Ca^2+ ^influx through P2X receptors or other Ca^2+ ^influx pathways is not required for the increased resensitization of the ATP response that is mediated by bradykinin.

To investigate the role of phosphorylation in the resensitization of GPCR/P2Y in response to bradykinin, we applied the PKC activator phorbol 12-myristate 13-acetate (PMA, 1 μM) for 10 minutes prior to the application of bradykinin (15 minutes before the first ATP application). Treatment with PMA significantly (p <0.001) reduced the peak amplitude of the first ATP response in both bradykinin treated and untreated cells by about 40%, with no significant difference between groups (Figures 3A). PMA treatment also significantly (p < 0.001) reduced resensitization of the ATP response in both groups. However, the bradykinin treated cells still showed significantly (p < 0.05) greater resensitization as compared with untreated cells (Figures 2A-B). These results indicate that activation of PKC significantly inhibits ATP mediated Ca^2+ ^signaling and reduces resensitization of the response to ATP. However, the effect of bradykinin on the resensitization of the ATP response continues to occur following activation of PKC by PMA.

The role of phosphatases in the GPCR/P2Y response was tested with okadaic acid (40 nM), a protein phosphatase 1 and 2A inhibitor. Okadaic acid did not have a significant effect on the initial response to ATP in bradykinin-treated and control cells (Figures 3A-B). However, okadaic acid blocked the bradykinin -evoked increase in resensitization of the second response to ATP (Figures 2A-B).

To determine the potential involvement of the PI3K pathway in the resensitization response to bradykinin, we applied the PI3K inhibitor LY294002 (50 μM) to both control and bradykinin -treated cells. LY294002 significantly (p < 0.001) inhibited both the initial response to ATP (Figures 3A-B) as well as the resensitization of the response to the second application of ATP. Treatment with LY294002 also inhibited the effect of bradykinin on resensitization of the ATP response. (Figures 2A-B)

## Discussion

Purinergic receptor-mediated signaling is critically important for the function of many cell types [1,2], but relatively little is known about the process by which purinergic signaling interacts with other types of receptor-mediated signaling. Given the potential importance for this kind of interaction, especially in glial tumors, we investigated the role of bradykinin signaling on purinergic signaling in the human glioma cell line.

In U87 astrocytoma cells, the preincubation with bradykinin had no significant effect on the peak amplitude and area under the curve (AUC) in the first response to ATP. However both peak amplitude and AUC of the response to second application of ATP were significantly increased by bradykinin. That this effect was blocked by the bradykinin the specific antagonist HOE 140, confirm that the resensitization phenomenon we observed is mediated by the activation of bradykinin receptors.

The calcium response of U87 cells to ATP is mediated primarily by P2Y purinergic receptors, as shown by the persistence of the response in zero Ca^2+ ^solution, as well as the complete inhibition of the response in cells treated with the PLC inhibitor U73122. The persistence of the bradykinin -induced increase in resensitization of the Ca^2+ ^response to ATP in the absence of extracellular Ca^2+ ^indicates that the entry of calcium through P2X receptors or other Ca^2+ ^influx pathways is not required for the effect of bradykinin on resensitization. Because bradykinin activates PKC, we investigated the effects of the PKC activator phorbol ester on ATP mediated calcium signaling and its modulation by bradykinin. Similar to the reports of others [34], we found that treatment with phorbol ester significantly reduced the initial response to ATP, and increased desensitization of the response. However, the increased resensitization of the ATP response in bradykinin treated cells was maintained, suggesting that PKC did not play a primary role in the bradykinin effect.

The resensitization effect of bradykinin was abolished by the phosphatase 1 and 2A inhibitor okadaic acid, consistent with a key role for dephosphorylation of P2Y purinergic receptors in the increased resensitization induced by bradykinin. Other investigators [34-36] have also found that phophatases are important for the resensitization of P2Y purinergic receptors, and their blockage with okadaic acid decreases receptor resensitization [36].

Our additional finding that the bradykinin effect was prevented by inhibition of PI3K with LY294002 is consistent with previous reports of a role for PI3K in resensitization of P2Y purinergic receptors in guinea pig Muller cells [37]. PI3K has been found to mediate phosphorylation and translocation of phospholipase C-gamma and resultant IP3 production in response to activation of P2Y purinergic receptors - this could be one mechanism by which PI3 kinase inhibitors inhibit both the initial response to ATP and the resensitization of P2Y purinergic receptors [38].

Resensitization of GPCR's may occur both through re-coupling of receptors to G proteins, as well as agonist-dependent endocytosis of the receptor to endosomal compartment followed by dephosphorylation and recycling back to the membrane as a functional receptor [18]. The U87 cells mainly express P2Y1 purinergic receptors [39] and it has recently been reported that agonist-mediated endocytosis of P2Y1 purinergic receptors occurs over a period of 30 minutes, with recycling of endocytosed receptors occurring over a period of approximately 60 minutes [40]. The duration of this process is therefore significantly greater than the 1 minute time period over which we observed increased resensitization of the ATP response in bradykinin treated cells. Thus, the effect of bradykinin that we observed is more likely to be due to a re-coupling of the receptor to G protein prior to recycling through the endosomal compartment.

The effects of bradykinin on ATP-mediated signaling could play multiple functional roles in the nervous system. B2 receptors are highly expressed on glioma cells, and the expression of these receptors may be correlated with tumor grade [29], suggesting that they may play a role in the abnormal growth and migration of these cells [28]. P2Ypurinergic receptors have also been implicated in the growth and migration of glial tumors [41-43]. Thus, potentiation of purinergic receptor signaling by bradykinin could play a role in CNS tumor formation and progression.

B2 receptors have also been a focus of investigation and potential therapies because of their role in opening the blood-tumor-barrier [44]. P2Y purinergic receptors are also highly expressed at the glio-vascular interface, suggesting that an interaction between bradykinin and P2Y purinergic receptors could play a role in the interaction of glioma cells with the vasculature [45]. Bradykinin is also well known as an important inflammatory mediator, with potential roles in the response of the brain to head injury, encephalitis, and ischemia [46]. Significant quantities of ATP may be released in association with these same events, which therefore represent other settings in which interaction between bradykinin and ATP-mediated signaling may be important [47]. Finally, both B2 and P2Y purinergic receptors may be involved in pain transmission. Bradykinin released in the setting of tissue damage or inflammation could therefore influence nociception by increased resensitization of P2Y purinergic receptors [48,49].

## Conclusions

Our results indicate that bradykinin-evoked signaling has a significant influence on purinergic receptor signaling via the PI3 kinase pathway. Potentiation of purinergic signaling by increasing receptor resensitization may represent an important mechanism by which bradykinin modulates glial cell function.

## Abbreviations

[Ca^2+^]_i_: intracellular calcium concentration; ATP: adenosine 5' triphosphate; PKC: protein kinase C; PMA: phorbol12-myristate 13-acetate; PI3K: Phosphoinositide 3-kinase.

## Competing interests

The authors declare that they have no competing interests.

## Authors' contributions

HELV and LBP carried out all experiments; KCB and AC contributed to the writing of the manuscript and participated in experimental designs. All authors read and approved the final manuscript.
